# Severe COVID-19 patients exhibit an ILC2 NKG2D^+^ population in their impaired ILC compartment

**DOI:** 10.1038/s41423-020-00596-2

**Published:** 2020-12-14

**Authors:** Alejandra Gomez-Cadena, Laurie Spehner, Marie Kroemer, Myriam Ben Khelil, Kevin Bouiller, Grégory Verdeil, Sara Trabanelli, Christophe Borg, Romain Loyon, Camilla Jandus

**Affiliations:** 1 Department of Pathology and Immunology, University of Geneva, Geneva, Switzerland, and Ludwig Institute for Cancer Research, Lausanne, Switzerland; 2 University of Bourgogne Franche-Comté, INSERM, EFS BFC, UMR1098, RIGHT Interactions Greffon-Hôte-Tumeur/Ingénierie Cellulaire et Génique, F-25000 Besançon, France; 3 Department of Pharmacy, University Hospital of Besançon, F-25000 Besançon, France; 4 Department of Infectious Disease, University Hospital of Besançon, F-25000 Besançon, France; 5 UMR-CNRS 6249 Chrono-Environment, Université Bourgogne Franche-Comté, F-25000 Besançon, France; 6 Department of Oncology, UNIL-CHUV, University of Lausanne, 1066 Epalinges, Switzerland; 7 Department of Medical Oncology, University Hospital of Besançon, F-25000 Besançon, France

**Keywords:** Innate lymphoid cells, Infection

Severe acute respiratory syndrome coronavirus 2 (SARS-CoV-2) is responsible for the current COVID-19 disease pandemic. In some patients, the symptoms are mild, and a fraction of SARS-CoV-2-infected individuals develop severe illness with a high fatality rate due to lung damage and acute respiratory distress syndrome.^1^

Innate lymphoid cells (ILCs) are a recently identified type of effector immune cells that rapidly sense environmental stimuli and participate in early immune responses by promptly secreting large amounts of cytokines.^2^ The ILC2 subpopulation was shown to mediate Type 2 responses and to recruit eosinophils during viral lung infections upon the release of alarmins (e.g., IL-33) by damaged epithelial cells.^3–5^ ILC2s were also shown to participate in the termination of inflammatory responses and tissue repair by amphiregulin secretion. In addition, ILC2s are critical in the early phases of allergic lung inflammation, including that induced by the protease allergen papain.^6^ Based on the essential function of the papain-like protease PLpro in regulating SARS-CoV-2^7^ (Fig. 1a) and the severe lung damage caused by this virus, we sought to investigate the potential involvement of ILC2s in immune responses to COVID-19.

**Fig. 1:**
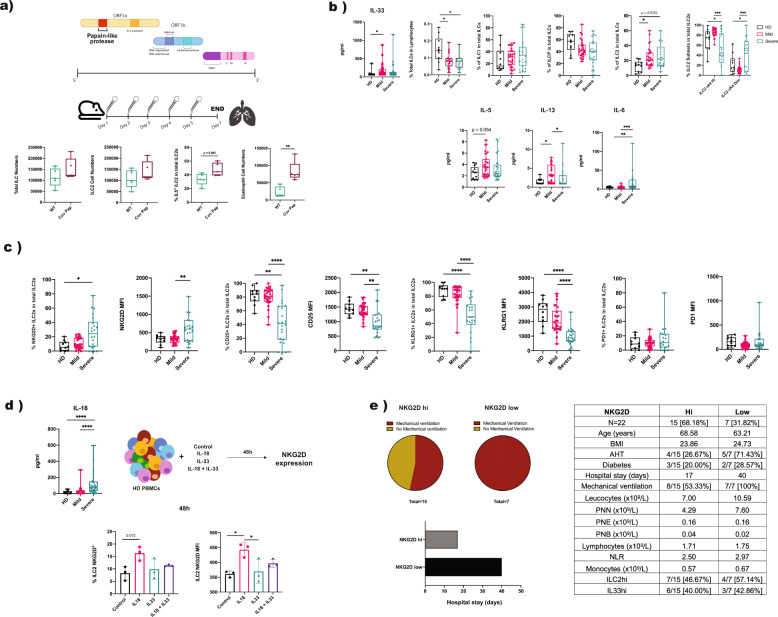
ILC2 increases and NKG2D expression in COVID-19 patients. **a** Lung cell numbers (total ILCs, ILC2s, and eosinophils) and the frequencies of IL-5^+^ ILC2s in WT animals treated intranasally with SARS-CoV-2 PLpro vs those in untreated animals. **b** Percentages of total ILCs and ILC subsets in PBMCs in HDs vs those in COVID-19 patients and serum cytokine levels in HDs vs COVID-19 patients. **c** Phenotyping of ILC2s in PBMCs of HDs and mild and severe COVID-19 patients. Percentages of positive ILC2s and the mean fluorescence intensity (MFI) of NKG2D, CD25, KLRG1, and PD-1 are shown. **d** Serum levels of IL-18 in HDs vs those in COVID-19 patients (upper part) and NKG2D expression and the MFI in HD PBMCs after 48 h of in vitro cytokine stimulation with hrIL-18, hrIL-33 or both vs those in unstimulated cells. **e** Clinical characteristics of severely ill COVID-19 patients classified as NKGD2^hi^ and NKGD2^low^ based on the median expression of NKG2D on ILC2s in the total patient cohort

As an initial proof of concept, we intranasally administered the papain-like protease PLpro in wild-type animals for 5 consecutive days (Fig. 1a). We observed a rapid increase in the total ILC and ILC2 numbers in the lungs. The percentage of IL-5^+^ ILC2s was increased upon papain challenge, which was concomitant with increased eosinophil infiltration (Fig. 1a) and compatible with PLpro-dependent ILC2 triggering.

Based on these observations, we next explored a retrospective cohort of 60 COVID-19 convalescent patients (mild *n* = 30, severe *n* = 30) previously described in Kroemer et al.^8^ Since plant-derived papain is known to act by stimulating epithelial cells to secrete IL-33,^9^ we hypothesized a similar mechanism for PLpro and quantified IL-33 in the sera of the patients. We observed a significant increase in IL-33 in COVID-19 patients compared to that in healthy donors (HDs), suggesting that in humans, PLpro might drive alarmin secretion (Fig. 1b). Next, by performing multiparametric flow cytometry-based immune monitoring of circulating ILCs, defined as Lin^−^CD127^+^ cells, we detected a reduction in total ILCs in severely ill patients, with a significant relative increase in the ILC2 subpopulation but no significant changes in other subpopulations (Fig. 1b). The proportions of cKit^high^ and cKit^dim^ ILC2s were comparable between HDs and mild COVID-19 patients. However, the cKit^dim^ subset was expanded in severe patients, which was compatible with an increase in fully mature ILC2s. Furthermore, we observed overall low but elevated levels of the Type 2 cytokines IL-5 and IL-13 in patients compared to those in HDs (Fig. 1b), while Type 1 and Type 17 cytokine levels were comparable across cohorts (data not shown). In line with the current literature, IL-6 levels were also increased in severe COVID-19 patients.

Upon activation, ILC2s modulate their phenotype by up/downregulating cell surface proteins. Therefore, we screened the expression of activating and inhibitory receptors on ILC2s in mild and severe patients. The ILC2s in severe patients showed an increase in the NKG2D^+^ population compared to those in mild patients and controls and a significant decrease in CD25 and KLRG1 (Fig. 1c). No differences in NKG2D, KLRG1, or CD25 expression were observed in ILC1s or ILCPs in patients (data not shown). The levels of other markers, such as PD-1, NKG2A, and NKp46, were similar on ILC2s from HDs and patients.

Of note, NKG2D, which is the activating C-type lectin-like molecule abundantly expressed by cytotoxic NK cells, has not been previously reported on ILC2s. However, its expression is known to be induced in NK cells by IL-33 and other members of the IL-1 family of cytokines, such as IL-18. Notably, serum IL-18 levels were significantly higher in severe COVID-19 patients than in patients with mild illness and HDs (Fig. 1d). Furthermore, ILC2s were previously reported to express IL-18R in the skin, lung and bone marrow^10^ and to react to IL-18 produced by Type 2 cytokine secretion. To verify whether NKG2D expression in ILC2s can be induced by elevations of the IL-33 or IL-18 concentrations in COVID-19 patients (Fig. 1b, d), we stimulated HD peripheral blood mononuclear cells (PBMCs) in vitro with recombinant human (rh) IL-33 or rhIL-18 alone or in combination for 48 h and monitored the ILC2 phenotype. We observed an increase in NKG2D expression in ILC2s exposed to IL-18 (Fig. 1d) but not in ILC2s exposed to IL-33, suggesting a direct link between IL-18 and NKG2D^+^ ILC2s in severe COVID-19 patients. To explore the potential clinical relevance of NKG2D^+^ ILC2s in anti-COVID-19 immune responses, we stratified patients based on the median expression of NKG2D on ILC2s (Fig. 1e). We observed a significantly reduced proportion of patients requiring mechanical ventilation in the severe group with high numbers of NKG2D^+^ ILC2s, indicating the protective role of this cell subset in the response against the virus. In line with this finding, the hospitalization length was drastically reduced in these patients (Fig. 1e).

Overall, our study shows an increase in ILC2s in COVID-19 patients in parallel with elevated serum Type 2 cytokine levels. These anti-inflammatory mediators might be produced particularly by NKG2D^+^ ILC2s upon engagement of the NKG2D receptor with its ligands, which are known to be upregulated on infected cells in the context of viral diseases.

## Supplementary information

Supplemenary material
