# Supplementary material for: Severe COVID-19 patients exhibit an ILC2 NKG2D+ population in their impaired ILC compartment
Source: Cell Mol Immunol. 2020 Dec 14;18(2):484–6. doi: 10.1038/s41423-020-00596-2 (PMC7734385; doi:10.1038/s41423-020-00596-2)
Supplement: Supplementary file 1 — Supplemenary material [file 41423_2020_596_MOESM1_ESM.docx]

**Supplementary materials**

**Methods**

**Reagents Table**

| **Reagent** | Reference | Provider |
| --- | --- | --- |
| Anti-human CD14-FITC | 130-110-518 | Miltenyi |
| Anti-human CD15-FITC | 130-113-484 | Miltenyi |
| Anti-human CD16-FITC | 130-113-392 | Miltenyi |
| Anti-human CD20-FITC | 130-111-337 | Miltenyi |
| Anti-human CD3-FITC | 130-113-138 | Miltenyi |
| Anti-human CD33-FITC | 130-111-018 | Miltenyi |
| Anti-human CD34-FITC | 130-113-178 | Miltenyi |
| Anti-human CD4-FITC | 130-114-531 | Miltenyi |
| Anti-human CD56-FITC | 130-114-549 | Miltenyi |
| Anti-human CD8-FITC | 130-110-677 | Miltenyi |
| Anti-human CD94-FITC | 130-098-971 | Miltenyi |
| Anti-human FcεRIα-FITC | 130-110-726 | Miltenyi |
| Anti-human CD203c-FITC | 324614 | Biolegend |
| Anti-human NKG2D-BV786 | 320830 | Biolegend |
| Anti-human CD25-BV510 | 302640 | Biolegend |
| Anti-human cKIT-BV605 | 313218 | Biolegend |
| Anti-human KLRG1-PE | 12-9488-41 | e-Bioscience |
| Anti-human CD127-PE-Dazzle | 351336 | Biolegend |
| Anti-human CRTH2-APC | 350104 | Biolegend |
| Recombinant hIL-18 | rcyec-hil18 | Invivogen |
| Recombinant hIL-33 | AG-40B-0160-C100 | Adipogen |
| Recombinant SARS-CoV-2 Papain-like Protease | E-611-050 | R&D Systems |
| DNAseI | 18047-019 | Invitrogen |
| Liberase TL | 540102001 | Roche |
| Recombinant mIL-33 | 580504 | Biolegend |
| Recombinant mIL-25 | 587304 | Biolegend |
| Anti-mouse CD3 FITC | 130-119-798 | Miltenyi |
| Anti-mouse CD5 FITC | 130-102-574 | Miltenyi |
| Anti-mouse CD8 FITC | 130-118-468 | Miltenyi |
| Anti-mouse CD19 FITC | 130-105-171 | Miltenyi |
| Anti-mouse B220 FITC | 130-118-462 | Miltenyi |
| Anti-mouse Ter119 FITC | 130-117-538 | Miltenyi |
| Anti-mouse FcεRIα FITC | 130-102-264 | Miltenyi |
| Anti-mouse DX5 FITC | 130-102-258 | Miltenyi |
| Anti-mouse TCRb FITC | 130-104-812 | Miltenyi |
| Anti-mouse TCR γ/δ FITC | 130-104-015 | Miltenyi |
| Anti-mouse CD45-A700 | 109822 | Biolegend |
| Anti-mouse CD90.3-BV605 | 202537 | Biolegend |
| Anti-mouse ST2-APC | 17-9335-80 | Invitrogen |
| Anti-mouse KLRG1-PECy7 | 138416 | Biolegend |
| Anti-mouse GATA3-Percp.Cy5.5 | 46-9966-42 | Invitrogen |
| Anti-mouse CD11b-BV650 | 101259 | Biolegend |
| Anti-mouse CD11c-BV711 | 117349 | Biolegend |
| Anti-mouse MHCII-PE | 116408 | Biolegend |
| Anti-mouse Ly6C-APCcy7 | 47-5932-80 | eBiosciences |
| Anti-mouse Ly6G-PE-TexasRed | 127647 | Biolegend |
| Anti-mouse SiglecF-BV421 | E502440 | BD |
| Anti-mouse IL13-PEefluor610 | 4311635 | eBiosciences |
| Anti-mouse IL4-BV711 | 504133 | Biolegend |
| Anti-mouse and human IL5-BV421 | 504311 | Biolegend |
| Golgi-Plug | 555029 | BD |
| Golgi-Stop | 554724 | BD |
| LIVE/DEAD™ Fixable Green Dead Cell Stain Kit | L23101 | Thermo Fisher |

**Samples Collection**

Patients, either hospitalized or seen in consultation with a biologically proven diagnosis of COVID-19 were included. According to WHO guidance, laboratory confirmation of SARS-Cov-2 was defined as a positive result of real-time reverse transcriptase–polymerase chain reaction (RT-PCR) assay of nasopharyngeal swabs and/or lower respiratory tract aspirates for patients hospitalized in intensive care unit. Patients were classified in 2 groups according WHO guidance^1^: mild illness (patients with uncomplicated upper respiratory tract viral infection), and severe pneumonia (patients with fever or suspected respiratory infection, plus one of the following: respiratory rate > 30 breaths/min; severe respiratory distress; or SpO2 ≤ 93.0% on room air).

Peripheral blood samples from patients were collected at least 21 days after the first positive SARS-CoV-2 PCR at the Departments of infectious disease or intensive care unit at the University Hospital of Besançon (France) from April to May 2020. The COV-CREM study is registered with ClinicalTrials.gov (Identifier: NCT04365322). This study is carried out in accordance with GCP-ICH-6 and conducted in a single university-affiliated hospital. Eligible patients were screened during hospital stay or medical consultation and received all information related to the study. Oral informed consent was obtained by investigators before inclusion. Patients’ characteristics are details in Kroemer et al.^2^.

Peripheral blood products were collected from anonymous healthy donors (HDs) at the Etablissement Français du Sang (EFS) (Besançon, France) after the signature of informed consent and following the EFS guidelines. The HDs selected for this study, were included between April 2014 and September 2019, prior of SARS-CoV-2 epidemic. Peripheral blood mononuclear cells (PBMC) from patients and HDs were isolated by density centrifugation on Ficoll gradient (Eurobio).

PBMC of HDs and patients were cryopreserved in CryoStor (CS10 and CS5) cell preservation media (Sigma-Aldrich) and were conserved in nitrogen for flow cytometry. Serum from HDs and patients were collected and conserved at -80°C for cytokine analyzes.

**Flow Cytometry**

Human-ILC2 *in vitro* stimulation: PBMCs were collected and washed with FACs buffer after incubation. Cells were stained with the green viability dye for 30 min at 4°C in the dark, then washed and stained with the antibody mix for 30 min at RT in the dark. The antibody mix was composed as follow: FITC Lineage (CD3, CD4, CD8, CD14, CD15, CD16, CD19, CD20, CD33, CD34, CD56, CD94, CD203c, FceRIa), NKG2D-BV786, CRTH2-APC, cKit-BV605, CD127-PE Dazzle, CD25-BV510 and KLRG1-PE.

Mouse-ILC2 *ex-vivo* and cytokine staining: Lung cells were collected right after processing or after stimulation and stained with the green viability dye for 30 min at 4°C in the dark. Then cells were washed and stained with the antibody mix for 30 min at RT in the dark. Cells were fixed and permeabilized using the FOXP3 Fix/Perm buffer set according to manufacturer’s indications and stained for transcription factors and cytokines intracellularly for 30 min at RT in the dark. Antibody mix for extracellular and intracellular staining contained the following antibodies: FITC lineage (CD3, CD5, CD8, CD45R, Ter119, FcεRIα, Dx5, TCRb, TCRγ/δ, CD19) CD45-A700, CD90.2-BV605, ST2-APC, KLRG1-PECy7, GATA3-PercpCy5.5, CD11b-BV650, CD11c-BV711, MHCII-PE, Ly6C-APCCy7, Ly6G-PE-TexasRed, SiglecF-BV421, IL-5-BV421, IL-4-BV711, IL-13-PEefluor610.

**Serum sample analysis**

Serum samples from HDS and patients were thawed and cytokines were quantified using the LEGENDplex Hu Th Cytokine panel 12-plex (Biolegend) and LEGEND Max Human IL-33 ELISA Kit (Biolegend).

**Papain Challenge**

WT C56BL/6 animals between 6 and 12 weeks were maintained in the conventional facility of the University of Lausanne (UNIL), Switzerland. This study was approved by the Veterinary Authority of the Swiss canton Vaud and performed in accordance with Swiss ethical guidelines and the protocols described in the animal experimentation license VD3455.

Mice were stimulated intranasally with 10ug of recombinant SARS-CoV-2-Papain-like Protease from R&D Systems in 20ul of PBS and the control group received 20ul of PBS alone. The papain was given for 5 consecutive days and 7 days after the first dose mice were euthanized to collect the lungs. Lungs were digested for 30 min at 37°C using Liberase TL 0.5mg/ml and DNAse I 4.5 U/ml in 1ml final volume/lung. After digestion cells were washed and red blood cell lysis performed. Cells were counted and half of them was stained as specified before for flow cytometry. The other half was *in vitro* stimulated overnight with 10ng/ml recombinant mIL-25 and 10ng/ml mIL-33 in 1ml final volume, Golgi plug and Golgi stop were added overnight and after incubation, cells were stained for intracellular cytokine production as specified above.

***In vitro* human ILC2 stimulation**

Human blood samples from healthy donors were collected at the local Blood Transfusion Center in Lausanne, Switzerland, under IRB approval (Ethics Committee, University Hospital of Lausanne – CHUV). Written informed consent was obtained from all healthy subjects, in accordance with the Declaration of Helsinki. Fresh anticoagulated blood diluted at a 1:2 ratio in PBS was layered on lymphoprep (ratio of diluted blood:lymphoprep 1.5:1). Mononuclear cells were isolated by density gradient centrifugation (1800 rpm, 20 min centrifugation without break, room temperature), washed twice and immediately cryopreserved in 90% fetal calf serum (FCS) and 10% DMSO. Freshly obtained PBMCs from 3 different HDs were cultured in at a cell density of 1x10^6^/ml in 2ml final volume in a 24 wells plate. Culture RPMI media was prepared with 8% human serum, 1% penicillin-streptomycin, 1% L-Glutamine, 1% non-essential amino acids, 1% Na pyruvate, kanamycin and 0.1% 2β-mercaptoethanol for the control conditions. The same media supplemented with recombinant hIL-18 (50ng/ml), hIL-33 (50ng/ml) or the combination of both was used to stimulate the cells over 48h at 37°C. After incubation cells were harvested and stained for flow cytometry analysis as described before.

**Data Analysis and Statistics**

Statistical analyses and plots were done using GraphPad Prism (version 8.1.1). For the comparison of groups, *p*-values were obtained using Kruskal-Wallis tests. For two groups analysis in the context of the mouse model Mann-Whitney tests were used. *p*-values were consider as follow: **p*<0.05; ***p*<0.01; ****p*<0.001;*****p*<0.0001. Boxplots represent the median and ranks, and the whiskers shows lowest and highest values.

**References**

1 Clinical management of severe acute respiratory infection when COVID-19 is suspected. https://www.who.int/publications-detail/clinical-management-of-severe-acute-respiratory-infection-when-novel-coronavirus-(ncov)-infection-is-suspected (accessed 15 May2020).

2 Kroemer M, Spehner L, Vettoretti L, Bouard A, Eberst G, Pili Floury S *et al.* COVID-19 patients display distinct SARS-CoV-2 specific T-cell responses according to disease severity. *J Infect* 2020; : 4816.
